# Supplementary material for: Association of Maternal Dietary Patterns during Pregnancy and Offspring Weight Status across Infancy: Results from a Prospective Birth Cohort in China
Source: Nutrients. 2021 Jun 15;13(6):2040. doi: 10.3390/nu13062040 (PMC8232115; doi:10.3390/nu13062040)
Supplement: Supplementary file 1 [file nutrients-13-02040-s001.zip › nutrients-1244746-supplementary.pdf]

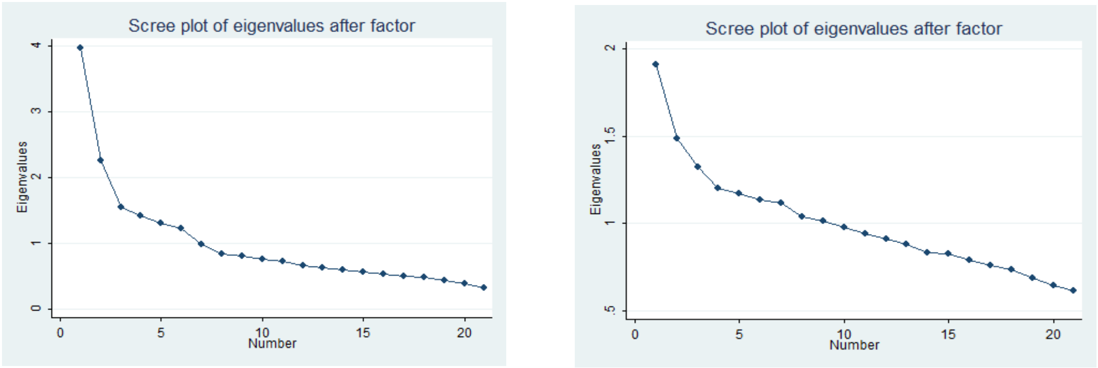

a. b.

Figure S1. Scree plot from exploratory factor analysis.

a: food Frequency Questionnaire; b: three-Day Food Diaries

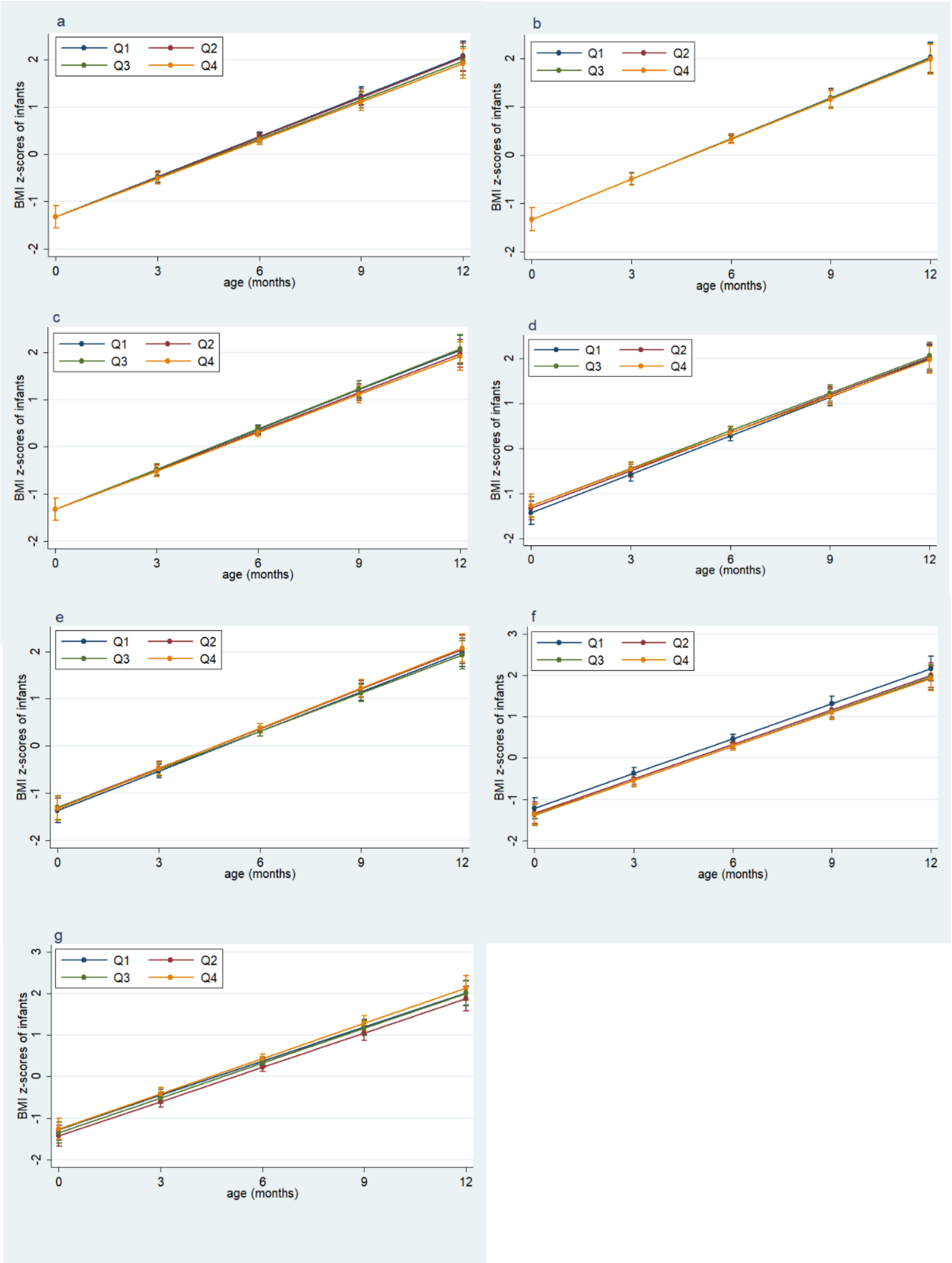

Figure S2. Predicted linear mixed effects models of BMI z-score and a) fish-seafood pattern (FFQ), b) protein rich pattern (FFQ), c) vegetable-fruit-rice pattern (FFQ), d) traditional pattern (TFD), e) sweet foods pattern (TFD), f) fried food-bean-dairy pattern (TFD), g) whole grain-seafood pattern (TFD) score quartiles of mothers as a function of age. Models are adjusted for other dietary patterns derived from the same dietary assessment tool (TFD or FFQ), pre-pregnancy BMI, age, parity, family income, education level, ethnicity, smoking status, total energy intake, physical activity and paternal BMI.

FFQ: food frequency questionnaire; TFD: three-day food diaries; BMI: body mass index; SD: standard deviation

Table S1. Characteristics of participants included and excluded in the present study.

Characteristics	Participants, mean ± SD or n (%)		
	Included	Excluded	*p*
**Mothers**			
Age at enrollment (Years)			0.001
<25	53 (5.7)	19 (5.3)	
25-29	399 (42.6)	147 (41.0)	
30-35	344 (36.7)	106 (29.5)	
>36	141 (15.1)	87 (24.2)	
Ethnicity			<0.001
Han	787 (84.0)	261 (72.7)	
Others	150 (16.0)	98 (27.3)	
Educational attainment			0.791
Middle school or below	76 (8.1)	30 (9.3)	
High school	146 (15.6)	55 (17.0)	
College	628 (67.0)	211 (65.3)	
Graduate or above	87 (9.3)	27 (8.4)	
Household income per year, CNY			0.460
<30,000	247 (26.4)	90 (27.9)	
30,000-<50,000	256 (27.3)	77 (23.8)	
50,000-<70,000	210 (22.4)	68 (21.1)	
≥70,000	224 (23.9)	88 (27.2)	
Parity			0.007
primiparous	739 (78.9)	231 (71.5)	
multiparous	198 (21.1)	92 (28.5)	
Smoking status			<0.001
Yes	4 (0.4)	40 (11.1)	
No	933 (99.6)	319 (88.9)	
Pre-pregnancy BMI category			0.796
<18.5, kg/m^2	123 (13.1)	43 (13.3)	
18.5-<24.0, kg/m^2	566 (60.4)	195 (60.4)	
24.0-<28.0, kg/m^2	186 (19.9)	59 (18.3)	
≥28.0, kg/m^2	62 (6.6)	26 (8.1)	
Physical Activity			<0.001
<100 MET-hour/week	230 (24.6)	135 (37.6)	
100 to <200 MET-hour/week	527 (56.2)	153 (42.6)	
≥200 MET-hour/week	180 (19.2)	71 (19.8)	
Energy intake			<0.001
<2100 Kcal/d	576 (61.5)	117 (32.6)	
≥2100 Kcal/d	361 (38.5)	242 (67.4)	
**Fathers**			
BMI at enrollment （n=903）			0.643
<18.5, kg/m^2	22 (2.4)	8 (2.6)	
18.5-<24.0, kg/m^2	344 (38.1)	111 (35.9)	

24.0-<28.0, kg/m^2	341 (37.8)	129 (41.8)	
≥28.0, kg/m^2	196 (21.7)	61 (19.7)	
**Infants**			
Sex			0.930
Male	447 (47.7)	155 (48.0)	
Female	490 (52.3)	168 (52.0)	

SD: standard deviation; CNY, Chinese Yuan; BMI, body mass index

Table S2. Spearman correlation between dietary patterns driven from food frequency questionnaires and three-day food diaries

Dietary patterns driven from TFD	Dietary patterns driven from FFQ					
	Fish-seafood pattern (FFQ)		Protein rich pattern (FFQ)		Vegetable-fruit-rice pattern (FFQ)	
	ϱ	*p*	ϱ	*p*	ϱ	*p*
**Traditional pattern (TFD)**	0.013	0.700	0.000	0.991	0.131	<0.001
**Sweet foods pattern (TFD)**	-0.058	0.075	-0.068	0.038	0.063	0.055
**Fried food-bean-dairy pattern (TFD)**	-0.026	0.434	0.008	0.806	-0.083	0.011
**Whole grain-seafood pattern (TFD)**	-0.013	0.688	0.023	0.483	-0.042	0.205

FFQ: food frequency questionnaire; TFD: three-day food diaries; BMI: body mass index; SD: standard deviation

Table S3. Associations of maternal dietary pattern quartiles with predicted WFL z-scores at birth, 3, 6, 8, 12 months in the "Born in Shenyang" Cohort.

Maternal dietary patterns	n	Mean difference in predicted WFL z-score, SD units									
		Birth		3 months		6 months		8 months		12 months	
		β	95% CI	β	95% CI	β	95% CI	β	95% CI	β	95% CI
**Food Frequency Questionnaire**											
Fish-seafood pattern (FFQ)											
Q1	234	ref	ref	ref	ref	ref	ref	ref	ref	ref	ref
Q2	231	0.05	(-0.13, 0.22)	0.03	(-0.12, 0.18)	0.01	(-0.13, 0.16)	-0.01	(-0.17, 0.16)	-0.02	(-0.23, 0.18)
Q3	235	0.09	(-0.09, 0.28)	0.06	(-0.10, 0.22)	0.03	(-0.12, 0.18)	-0.01	(-0.18, 0.17)	-0.04	(-0.25, 0.17)
Q4	237	0.16	(-0.03, 0.36)	0.10	(-0.06, 0.27)	0.04	(-0.12, 0.21)	-0.02	(-0.21, 0.17)	-0.08	(-0.31, 0.15)
Protein rich pattern (FFQ)											
Q1	237	ref	ref	ref	ref	ref	ref	ref	ref	ref	ref
Q2	231	-0.16	(-0.33, 0.01)	-0.15	(-0.29, -0.01)	-0.14	(-0.28, 0.01)	-0.13	(-0.29, 0.04)	-0.11	(-0.32, 0.09)
Q3	235	-0.16	(-0.34, 0.03)	-0.14	(-0.30, 0.02)	-0.12	(-0.27, 0.03)	-0.10	(-0.28, 0.07)	-0.09	(-0.30, 0.13)
Q4	234	-0.15	(-0.35, 0.05)	-0.15	(-0.32, 0.02)	-0.15	(-0.31, 0.02)	-0.15	(-0.33, 0.04)	-0.14	(-0.38, 0.09)
Vegetable-fruit-rice pattern (FFQ)											
Q1	229	ref	ref	ref	ref	ref	ref	ref	ref	ref	ref
Q2	236	0.14	(-0.03, 0.30)	0.10	(-0.04, 0.24)	0.07	(-0.07, 0.20)	0.03	(-0.13, 0.19)	0.00	(-0.20, 0.19)
Q3	236	0.18	(0.02, 0.35)	0.17	(0.03, 0.31)	0.16	(0.02, 0.30)	0.14	(-0.01, 0.30)	0.13	(-0.06, 0.33)
Q4	236	0.18	(0.01, 0.35)	0.13	(-0.01, 0.28)	0.09	(-0.06, 0.23)	0.05	(-0.12, 0.21)	0.00	(-0.20, 0.20)
**Three-day food dairies**											
Traditional pattern (TFD)											
Q1	233	ref	ref	ref	ref	ref	ref	ref	ref	ref	ref
Q2	236	0.04	(-0.13, 0.20)	0.04	(-0.10, 0.17)	0.04	(-0.10, 0.17)	0.03	(-0.12, 0.19)	0.03	(-0.16, 0.23)
Q3	231	0.14	(-0.04, 0.30)	0.11	(-0.04, 0.25)	0.08	(-0.06, 0.22)	0.05	(-0.11, 0.22)	0.03	(-0.17, 0.23)
Q4	237	0.08	(-0.10, 0.26)	0.04	(-0.12, 0.20)	0.01	(-0.15, 0.16)	-0.03	(-0.21, 0.14)	-0.07	(-0.28, 0.14)

Sweet foods pattern (TFD)											
Q1	232	ref	ref	ref	ref	ref	ref	ref	ref	ref	ref
Q2	238	0.08	(-0.09, 0.24)	0.08	(-0.06, 0.22)	0.08	(-0.06, 0.21)	0.07	(-0.08, 0.23)	0.07	(-0.12, 0.27)
Q3	230	0.06	(-0.11, 0.22)	0.03	(-0.11, 0.18)	0.01	(-0.13, 0.15)	-0.01	(-0.17, 0.15)	-0.03	(-0.23, 0.16)
Q4	237	0.02	(-0.15, 0.19)	0.04	(-0.10, 0.18)	0.06	(-0.08, 0.20)	0.08	(-0.08, 0.24)	0.10	(-0.09, 0.30)
Fried food-bean-dairy pattern (TFD)											
Q1	231	ref	ref	ref	ref	ref	ref	ref	ref	ref	ref
Q2	230	-0.02	(-0.19, 0.15)	-0.06	(-0.20, 0.08)	-0.10	(-0.24, 0.03)	-0.15	(-0.30, 0.01)	-0.19	(-0.38, 0.01)
Q3	233	-0.12	(-0.29, 0.04)	-0.15	(-0.29, -0.01)	-0.18	(-0.32, -0.04)	-0.20	(-0.36, -0.04)	-0.23	(-0.43, -0.03)
Q4	243	-0.12	(-0.29, 0.05)	-0.15	(-0.30, -0.01)	-0.19	(-0.33, -0.04)	-0.22	(-0.38, -0.06)	-0.26	(-0.45, -0.06)
Whole grain-seafood pattern (TFD)											
Q1	235	ref	ref	ref	ref	ref	ref	ref	ref	ref	ref
Q2	237	-0.19	(-0.36, -0.03)	-0.18	(-0.32, -0.04)	-0.16	(-0.30, -0.03)	-0.16	(-0.31, 0.00)	-0.14	(-0.33, 0.05)
Q3	231	-0.08	(-0.25, 0.08)	-0.06	(-0.20, 0.08)	-0.04	(-0.18, 0.10)	-0.02	(-0.18, 0.14)	0.00	(-0.20, 0.20)
Q4	234	-0.05	(-0.22, 0.12)	-0.01	(-0.15, 0.14)	0.03	(-0.10, 0.17)	0.08	(-0.08, 0.23)	0.12	(-0.08, 0.31)

Adjusted for other dietary patterns derived from the same dietary assessment tool (TFD or FFQ), pre-pregnancy BMI, age, parity, family income, education level, ethnicity, smoking status, total energy intake, physical activity, paternal BMI

WFL: weight for length; Q1: quartile 1; Q2: quartile 2; Q3: quartile 3; Q4: quartile 4; OR: odd ratio; CI: confidence interval; FFQ: food frequency questionnaire; TFD: three-day food diaries; BMI: body mass index

Table S4. Associations of maternal dietary pattern scores during pregnancy, in quartiles, with risk of being overweight/obesity across the infancy, with further adjustment for gestational weight gain.

Maternal Dietary Patterns during pregnancy	Risk of being overweight/obesity across infancy (ref.=non overweight/obesity, n=903)				
	Q1 Reference	Q2 OR (95% CI)	Q3 OR (95% CI)	Q4 OR (95% CI)	P for trend
**Food Frequency Questionnaire**					
Fish-seafood pattern (FFQ)	1.00	0.91 (0.53, 1.57)	1.13 (0.66, 1.94)	0.78 (0.42, 1.46)	0.439
Protein rich pattern (FFQ)	1.00	0.65 (0.38, 1.12)	0.51 (0.28, 0.93)	0.78 (0.42, 1.45)	0.441
Vegetable-fruit-rice pattern (FFQ)	1.00	1.37 (0.75, 2.50)	1.77 (1.02, 3.08)	1.54 (0.84, 2.84)	0.149
**Three-day food dairies**					
Traditional pattern (TFD)	1.00	0.79 (0.44, 1.45)	1.06 (0.61, 1.84)	0.73 (3.71, 1.44)	0.787
Sweet foods pattern (TFD)	1.00	1.53 (0.87, 2.71)	1.08 (0.56, 2.05)	1.47 (0.81, 2.66)	0.531
Fried food-bean-dairy pattern (TFD)	1.00	0.69 (0.42, 1.15)	0.55 (0.31, 0.98)	0.64 (0.35, 1.19)	0.427
Whole grain-seafood pattern (TFD)	1.00	0.71 (0.39, 1.30)	0.73 (0.42, 1.27)	0.91 (0.53, 1.57)	0.612

Adjusted for other dietary patterns derived from the same dietary assessment tool (TFD or FFQ), pre-pregnancy BMI, age, parity, family income, education level, ethnicity, smoking status, total daily calorie intake, physical activity, gestational weight gain, paternal BMI

Q1: quartile 1; Q2: quartile 2; Q3: quartile 3; Q4: quartile 4; OR: odd ratio; CI: confidence interval; FFQ: food frequency questionnaire; TFD: three-day food diaries; BMI: body mass index

Table S5. Associations of maternal dietary pattern scores during pregnancy, in quartiles, with BMI z-score across the infancy in the "Born in Shenyang" Cohort.

Maternal Dietary Patterns during pregnancy	Infant BMI z-score (n=937)				
	Q1 Reference	Q2 β (95% CI)	Q3 β (95% CI)	Q4 β (95% CI)	P for trend
**Food Frequency Questionnaire**					
Fish-seafood pattern (FFQ)					
Model 1	0.00	-0.04 (-0.17, 0.10)	-0.03 (-0.16, 0.11)	-0.04 (-0.17, 0.10)	0.678
Model 2	0.00	0.03 (-0.11, 0.18)	0.06 (-0.09, 0.21)	0.08 (-0.08, 0.24)	0.372
Model 3	0.00	-0.05 (-0.19, 0.09)	-0.03 (-0.16, 0.11)	-0.04 (-0.18, 0.10)	0.712
Model 4	0.00	0.01 (-0.14, 0.15)	0.04 (-0.11, 0.19)	0.05 (-0.11, 0.22)	0.479
Protein rich pattern (FFQ)					
Model 1	0.00	-0.17 (-0.30, -0.03)	-0.15 (-0.28, -0.01)	-0.16 (-0.30, -0.03)	0.037
Model 2	0.00	-0.19 (-0.33, -0.05)	-0.19 (-0.34, -0.04)	-0.21 (-0.37, -0.05)	0.022
Model 3	0.00	-0.15 (-0.28, -0.01)	-0.11 (-0.25, 0.03)	-0.13 (-0.27, 0.01)	0.123
Model 4	0.00	-0.16 (-0.30, -0.02)	-0.14 (-0.29, 0.01)	-0.17 (-0.36, -0.01)	0.072
Vegetable-fruit-rice pattern (FFQ)					
Model 1	0.00	0.05 (-0.08, 0.19)	0.12 (-0.02, 0.26)	0.02 (-0.12, 0.15)	0.623
Model 2	0.00	0.07 (-0.07, 0.20)	0.14 (0.00, 0.28)	0.05 (-0.09, 0.19)	0.403
Model 3	0.00	0.06 (-0.08, 0.19)	0.15 (0.01, 0.28)	0.06 (-0.07, 0.20)	0.230
Model 4	0.00	0.07 (-0.06, 0.21)	0.16 (0.03, 0.30)	0.09 (-0.05, 0.23)	0.145
**Three-day food dairies**					
Traditional pattern (TFD)					
Model 1	0.00	0.03 (-0.10, 0.17)	0.10 (-0.04, 0.23)	0.02 (-0.11, 0.16)	0.716
Model 2	0.00	0.04 (-0.10, 0.17)	0.08 (-0.05, 0.22)	0.01 (-0.13, 0.14)	0.731
Model 3	0.00	0.07 (-0.07, 0.21)	0.14 (-0.01, 0.29)	0.09 (-0.08, 0.26)	0.294
Model 4	0.00	0.06 (-0.08, 0.20)	0.11 (-0.04, 0.26)	0.05 (-0.12, 0.22)	0.267
Sweet foods pattern (TFD)					
Model 1	0.00	0.06 (-0.07, 0.20)	-0.03 (-0.16, 0.11)	0.01 (-0.13, 0.14)	0.781
Model 2	0.00	0.09 (-0.04, 0.23)	0.02 (-0.12, 0.15)	0.05 (-0.09, 0.18)	0.750
Model 3	0.00	0.06 (-0.08, 0.19)	-0.01 (-0.15, 0.13)	0.03 (-0.11, 0.17)	0.876
Model 4	0.00	0.08 (-0.05, 0.22)	0.03 (-0.11, 0.17)	0.06 (-0.08, 0.20)	0.814
Fried food-bean-dairy pattern (TFD)					
Model 1	0.00	-0.13 (-0.26, 0.01)	-0.18 (-0.32, -0.05)	-0.19 (-0.33, -0.06)	0.742
Model 2	0.00	-0.13 (-0.27, 0.01)	-0.19 (-0.33, -0.06)	-0.21 (-0.35, -0.07)	0.752
Model 3	0.00	-0.11 (-0.24, 0.02)	-0.17 (-0.31, -0.03)	-0.19 (-0.33, -0.05)	0.854
Model 4	0.00	-0.11 (-0.25, 0.02)	-0.18 (-0.31, -0.04)	-0.19 (-0.34, -0.05)	0.847
Whole grain-seafood pattern (TFD)					
Model 1	0.00	-0.16 (-0.29, -0.02)	-0.07 (-0.21, 0.07)	-0.02 (-0.15, 0.12)	0.825
Model 2	0.00	-0.14 (-0.28, -0.01)	-0.06 (-0.19, 0.08)	0.03 (-0.11, 0.16)	0.825

Model 3	0.00	-0.16 (-0.29, -0.02)	-0.07 (-0.21, 0.06)	0.02 (-0.11, 0.16)	0.455
Model 4	0.00	-0.14 (-0.27, -0.01)	-0.06 (-0.20, 0.08)	0.05 (-0.08, 0.19)	0.425

Model 1: crude model

Model 2: adjusted for other dietary patterns derived from the same dietary assessment tool (TFD or FFQ)

Model 3: adjusted for pre-pregnancy BMI, age, parity, family income, education level, ethnicity, smoking status, total energy intake, physical activity, paternal BMI

Model 4: model 2 + model 3

BMI: body mass index; Q1: quartile 1; Q2: quartile 2; Q3: quartile 3; Q4: quartile 4; CI: confidence interval; FFQ: food frequency questionnaire; TFD: three-day food diaries

Table S6. Associations of maternal dietary pattern scores during pregnancy, in quartiles, with WFL z-score across the infancy in the "Born in Shenyang" Cohort.

Maternal Dietary Patterns during pregnancy	Infant WFL z-score (n=937)				
	Q1 Reference	Q2 β (95% CI)	Q3 β (95% CI)	Q4 β (95% CI)	P for trend
**Food Frequency Questionnaire**					
Fish-seafood pattern (FFQ)					
Model 1	0.00	-0.03 (-0.17, 0.10)	-0.04 (-0.17, 0.10)	-0.03 (-0.16, 0.11)	0.769
Model 2	0.00	0.03 (-0.11, 0.18)	0.05 (-0.10, 0.20)	0.09 (-0.08, 0.25)	0.310
Model 3	0.00	-0.04 (-0.18, 0.10)	-0.03 (-0.17, 0.11)	-0.03 (-0.16, 0.11)	0.811
Model 4	0.00	0.01 (-0.13, 0.16)	0.03 (-0.12, 0.18)	0.06 (-0.10, 0.22)	0.432
Protein rich pattern (FFQ)					
Model 1	0.00	-0.16 (-0.30, -0.02)	-0.13 (-0.27, 0.010)	-0.16 (-0.29, -0.04)	0.047
Model 2	0.00	-0.20 (-0.34, -0.05)	-0.18 (-0.33, -0.03)	-0.21 (-0.37, -0.05)	0.022
Model 3	0.00	-0.14 (-0.27, 0.00)	-0.09 (-0.23, 0.05)	-0.12 (-0.25, 0.02)	0.176
Model 4	0.00	-0.16 (-0.30, -0.02)	-0.12 (-0.27, 0.03)	-0.16 (-0.32, 0.01)	0.091
Vegetable-fruit-rice pattern (FFQ)					
Model 1	0.00	0.06 (-0.08, 0.19)	0.12 (-0.01, 0.26)	0.04 (-0.10, 0.17)	0.446
Model 2	0.00	0.07 (-0.06, 0.21)	0.14 (0.01, 0.28)	0.07 (-0.07, 0.21)	0.266
Model 3	0.00	0.06 (-0.08, 0.19)	0.15 (0.01, 0.29)	0.08 (-0.06, 0.22)	0.152
Model 4	0.00	0.07 (-0.07, 0.20)	0.17 (0.03, 0.30)	0.10 (-0.04, 0.24)	0.092
**Three-day food dairies**					
Traditional pattern (TFD)					
Model 1	0.00	0.02 (-0.12, 0.15)	0.09 (-0.04, 0.23)	0.00 (-0.13, 0.14)	0.892
Model 2	0.00	0.02 (-0.12, 0.15)	0.08 (-0.06, 0.22)	-0.01 (-0.15, 0.13)	0.889
Model 3	0.00	0.05 (-0.09, 0.19)	0.13 (-0.02, 0.28)	0.07 (-0.10, 0.23)	0.430
Model 4	0.00	0.04 (-0.10, 0.18)	0.10 (-0.05, 0.25)	0.02 (-0.15, 0.19)	0.395
Sweet foods pattern (TFD)					
Model 1	0.00	0.09 (-0.05, 0.22)	-0.02 (-0.16, 0.12)	0.02 (-0.12, 0.15)	0.806
Model 2	0.00	0.11 (-0.02, 0.25)	0.02 (-0.12, 0.16)	0.05 (-0.08, 0.19)	0.798
Model 3	0.00	0.08 (-0.05, 0.21)	0.00 (-0.14, 0.13)	0.04 (-0.09, 0.18)	0.782
Model 4	0.00	0.10(-0.03, 0.24)	0.03 (-0.10, 0.17)	0.07 (-0.07, 0.21)	0.734
Fried food-bean-dairy pattern (TFD)					
Model 1	0.00	-0.09 (-0.22, 0.05)	-0.17 (-0.30, -0.03)	-0.18 (-0.31, -0.04)	0.994
Model 2	0.00	-0.09 (-0.23, 0.05)	-0.17 (-0.31, -0.04)	-0.19 (-0.33, -0.05)	0.993
Model 3	0.00	-0.07 (-0.20, 0.07)	-0.15 (-0.29, -0.02)	-0.16 (-0.30, -0.02)	0.984
Model 4	0.00	-0.07 (-0.21, 0.07)	-0.16 (-0.30, -0.02)	-0.17 (-0.31, -0.03)	0.987
Whole grain-seafood pattern (TFD)					
Model 1	0.00	-0.16 (-0.29, -0.02)	--0.05 (-0.19, 0.08)	-0.03 (-0.17, 0.10)	0.934
Model 2	0.00	-0.14 (-0.28, -0.01)	-0.04 (-0.18, 0.09)	0.01 (-0.13, 0.15)	0.927

Model 3	0.00	-0.16 (-0.29, -0.03)	-0.06 (-0.19, 0.08)	0.01 (-0.13, 0.15)	0.533
Model 4	0.00	-0.15 (-0.28, -0.01)	-0.05 (-0.18, 0.09)	0.04 (-0.10, 0.1)	0.512

Model 1: crude model

Model 2: adjusted for other dietary patterns derived from the same dietary assessment tool (TFD or FFQ)

Model 3: adjusted for pre-pregnancy BMI, age, parity, family income, education level, ethnicity, smoking status, total energy intake, physical activity, paternal BMI

Model 4: model 2 + model 3

WFL: weight for length; BMI: body mass index; Q1: quartile 1; Q2: quartile 2; Q3: quartile 3; Q4: quartile 4; CI: confidence interval; FFQ: food frequency questionnaire; TFD: three-day food diaries
